# Zinc in Well Water and Infant Mortality in Bangladesh: A Report from Gonoshasthaya Kendra

**DOI:** 10.3390/ijerph9010171

**Published:** 2012-01-10

**Authors:** Nicola Cherry, Corbett McDonald, Zafrullah Chowdhury

**Affiliations:** 1 Division of Preventive Medicine, University of Alberta, 5-30 University Terrace, 8303-112 Street, Edmonton, AB T6G 1K4, Canada; 2 National Heart and Lung Institute, Imperial College, South Kensington Campus, London SW7 2AZ, UK; Email: c.mcdonald@imperial.ac.uk; 3 Gonoshasthaya Kendra, Savar, Dhaka 1344, Bangladesh; Email: zaf.chowdhury@gmail.com

**Keywords:** zinc, drinking water, infant mortality, diarrhoea, Bangladesh

## Abstract

Zinc supplementation reduces the duration, severity and recurrence of diarrhoea in young children. This study examines whether zinc, found naturally in drinking water, reduced infant deaths from diarrhoea in rural Bangladesh. Information was compiled for births over two calendar years with follow-up for deaths within one year of birth. The study included 29,744 live births and 934 deaths in some 600 villages under the care of Gonoshasthaya Kendra (GK), grouped into 15 health centre regions within 12 upazillas. Individual matching of death to birth data was not possible, but information on exposures through well water and on potential confounders was available for each upazilla. Average concentration of zinc in well water, reported by the British Geological Survey, was grouped into high (>0.07 mg/L), moderate (0.020–0.070 mg/L) and low (<0.020 mg/L) concentrations. Odds ratios (OR) were calculated for zinc by age and cause of death. Zinc concentration was unrelated to all-cause mortality but a decrease in deaths from diarrhoea (N = 50) was seen in areas with high zinc (OR = 0.30; 95% CI 0.13–0.69). No relation to diarrhoeal deaths was found with other well contaminants (arsenic, manganese) having accounted for zinc. Upazillas with a high proportion of women without education had higher rates of death from diarrhea, but the decrease in risk with high zinc remained (OR adjusted = 0.41; 95% CI 0.20–0.84). It is concluded that exposure to zinc through drinking water may reduce risk of diarrhoeal deaths.

## 1. Introduction

In Bangladesh, contamination of drinking water with arsenic is widespread and health effects from arsenic exposure during pregnancy, childhood, and adult life have been widely studied in recent years. Much less attention has been paid to other drinking water contaminants, although some studies of the neurotoxic and reproductive effects of manganese have been reported. Information on well water contaminants was systematically collected in 1998–2000 by the Government of Bangladesh, together with the British Geological Survey (BGS), which analyzed water from 3,534 tube-wells from 61 of the 64 districts in Bangladesh [1]. In addition to arsenic and manganese, 18 other constituents were measured, including zinc. Zinc was of interest for three reasons. First, zinc deficiency has long been a concern for fetal health and a considerable number of trials have been conducted of the effects of zinc supplementation in pregnancy. A trial in Bangladesh did not show beneficial effects of supplementation [2], but a Cochrane review of 17 randomised controlled trials found a reduced number of preterm births in mothers receiving zinc (typically 20–30 mg/day) [3]. The second reason for interest in zinc arose from the use of zinc supplementation as treatment to reduce the duration and severity of diarrhoea once symptoms had appeared [4]. Infant mortality in Bangladesh, as elsewhere in the developing world, has historically reflected high rates of diarrhoea. Following a worldwide trend deaths from diarrhoea have been reduced in Bangladesh in recent years [5]. In Bangladesh this may reflect the increased access to relatively bacteria free water from tube wells as well as use of oral rehydration salts (ORS) and, most recently, zinc supplementation in diarrhoeal treatment. Third, there have been a significant number of trials (37 in the most recent meta-analysis) in which zinc supplementation has been used as an attempt to prevent rather that treat diarrhoea [6]. While a great deal of heterogeneity in results was found, it appeared that trials in Asia had been effective in preventing incident diarrhoea: none had looked at deaths specifically from diarrhoea. As part of our ongoing work with Gonoshasthaya Kendra (GK), an NGO with responsibility for health care of some 600 villages, information on infant mortality was available for villages in all Divisions of Bangladesh except Khulna and Sylhet. This information, initially analysed for the relationship of infant mortality to arsenic and manganese in well water [7], has been used in the present report to examine whether higher levels of zinc in drinking water were associated with a reduction in infant mortality, overall or from diarrhoea. 

## 2. Methods

The project was considered and approved by the University of Alberta Health Research Ethics Board and the GK ethics committee. Paramedics assigned to villages under the care of GK regularly visit all women of childbearing age, provide antenatal and postnatal care and routinely record detailed information on all pregnancies including data on maternal reproductive history, education, and socioeconomic factors [8]. They also carry out verbal autopsies on all infant deaths and record details including age (in days) and apparent cause. Information on all live births in GK villages in two years (Bangla 1409–1410, falling within 2001–2003 in the Western calendar) were available for this analysis together with information on all deaths at less than 12 months of age in babies born in these two years. Birth and death data from these villages were reported by 15 health centres, grouped geographically into 12 upazillas (also called thanas) which form the lowest administrative area within Bangladesh. Linkage between birth and death files at the individual level was not possible.

Concentrations of zinc were extracted from the BGS dataset, in which 7–14 wells had been sampled in each of the 12 upazillas. The mean concentration was taken to represent the exposure of all mothers giving birth in that upazilla and grouped into three levels, with four upazillas in each: high >0.07 mg/L; moderate 0.02–0.07 mg/L; low <0.02 mg/L. In general each upazilla included only one health centre but one, with mean concentration of 0.071 mg/L, contained two health centres and a second, with mean concentration on 0.020 mg/L, three centres.

### Statistical Methods

The numbers of births and deaths in each of the two years at each of 15 centres were extracted, giving 30 estimates of mortality rates as data points for the analysis. The death rate/1,000, age at death and cause of death were examined in relation to zinc concentration. Odds ratios were calculated using a maximum likelihood estimator, clustered within upazillas, with each unit of observation being the number of deaths from the number of live births in that upazilla. The blogit program within Stata [9] allows such a clustered analysis of grouped binomial data. The influence of possible covariates (concentrations of arsenic (<10: 10 < 50; ≥50 µg/L) and of manganese (<0.4: ≥0.4 mg/L)) in the well water and proportions of first pregnancies, mothers without formal education and low socioeconomic status among mothers giving birth in the centre were also examined. 

## 3. Results

There were 934 deaths within the first year of life amongst the 29,744 live births recorded, an infant mortality rate of 31.4/1,000. These included 404 deaths at <8 days, 205 at 8–28 days, and 325 at ≥29 days. Pneumonia was given as the most common cause of death at all ages, accounting for 458 deaths. Most deaths assigned to prematurity (N = 262) occurred within the first seven days and most deaths from diarrhoea (N = 50) at ≥29 days [7]. Concentrations of zinc in the 114 individual wells sampled by the BGS in these 12 upazillas ranged from none detected (ND)—0.45 mg/L. Means for the 12 upazillas ranged from 0.01–0.10 mg/L. Correlations between mean zinc concentration and either arsenic (r = 0.25, *p* = 0.36) or manganese (r = 0.30, *p* = 0.28) were low. Information on each of the upazillas is given in Table 1 (which represents a correction from similar data published earlier [7]), from which it can be seen that all-cause deaths/1,000 showed no trend with zinc concentration (high 30.1/1,000: moderate 33.8/1,000: low 31.6/1,000) but that deaths/1,000 from diarrhoea were lower in the high zinc group (0.72/1,000) than in the moderate (2.13/1,000) or low (2.38/1,000). Areas with low zinc tended to have somewhat more births to women of low socioeconomic status or with no education and appeared less likely to be first births. 

**Table 1:** Description of the upazillas included in the study.

Upazilla	Births	Deaths	Diarrhoeal deaths	Low social class	No Education	First birth	Zinc (mg/L)			Wells tested
	N *	% *	/1,000 *	% *	% *	% *	mean	SD	range	N
Bera	1,009.0	3.37	0.50	85.7	74.6	33.2	0.095	0.149	0.018–0.451	8
Parbatipur	1,773.5	2.00	0.84	86.1	84.0	22.6	0.086	0.053	0.020–0.162	8
Sripur	1,620.0	4.26	1.23	81.7	62.8	32.5	0.083	0.028	0.018–0.111	8
Gazipur Sadar	1,186.5	2.19	0.00	60.9	61.5	37.5	0.071	0.138	0.013–0.480	11
*Total high zinc*	*5,589.0*	*3.01*	**0.72**	*79.4*	*71.4*	*30.5*	*0.083*	*0.105*	*0.013*–*0.480*	*35*
Sirajganj Sadar	1,095.5	3.74	0.92	90.5	67.6	27.3	0.053	0.095	0.008–0.379	14
Saturia	742.5	2.63	3.37	74.9	70.1	34.7	0.041	0.063	0.004–0.211	10
Sonagazi	642.0	3.37	3.89	77.7	60.7	32.7	0.037	0.065	0.009–0.219	10
Savar	1,974.5	3.27	1.77	51.6	59.6	37.4	0.020	0.016	0.009–0.054	9
*Total moderate zinc*	*4,454.5*	*3.38*	**2.13**	*68.8*	*63.5*	*33.8*	*0.040*	*0.069*	*0.004*–*0.379*	*43*
Shibganj (N)	1,341.5	1.83	1.49	89.1	70.4	25.8	0.019	0.015	0.010–0.055	9
Coxs Bazar Sadar	482.5	4.15	1.04	83.0	63.5	35.9	0.015	0.004	0.006–0.019	7
Sherpur Sadar	1,861.0	4.22	3.76	89.7	88.5	28.0	0.013	0.003	0.007–0.017	12
Char Fasson	1,143.5	2.58	1.75	94.2	90.6	26.2	0.011	0.006	0.004–0.020	8
*Total low zinc*	*4,828.5*	*3.16*	**2.38**	*89.9*	*81.5*	*27.7*	*0.015*	*0.008*	*0.004*–*0.055*	*36*
Total	14,872.0	3.14	1.68	79.6	72.3	30.6	0.045	0.076	0.004–0.480	114

* mean of birth/death reports for all villages for 2 years.

Calculated odds ratios for zinc are shown in Table 2, confirming that the only relation unlikely to be due to chance was the decreased risk, specific to diarrhoea, with high zinc concentration (OR = 0.30; 95% CI 0.13–0.69). No dose-response relation was seen with all-cause mortality in any of the time periods examined, except for a non-significant trend to lower risk with increasing zinc in those who survive the first 28 days of life. Deaths ascribed to prematurity tended to be somewhat higher and deaths from pneumonia somewhat lower, in areas with ‘high’ zinc. Earlier analysis [7] has suggested that diarrhoeal deaths were somewhat elevated in those with exposure to arsenic 10 < 50 µg/L (OR = 2.14; 95% CI 1.07–4.29) but with a smaller OR at ≥50 µg/L (OR = 1.57; 95% CI 0.40–6.11). Having allowed for zinc there remained no significant relation of diarrhoeal deaths to arsenic (OR 10 < 50 µg/L =1.32 95% CI 0.76–2.31; ≥50 µg/L 1.72 95% CI 0.85–3.47), with <10 µg/L as the reference). The relation of diarrhoeal deaths to manganese concentration was not significant either in a bivariate analysis or after allowance for zinc (≥0.4 mg/L OR = 1.14 95% CI 0.64–2.03). No relationship was seen between deaths from diarrhoea and either low socioeconomic status or first births. Infants born in upazillas with a high proportion of births to mothers with no formal schooling were at greater risk of deaths attributed to diarrhoea, but inclusion of this factor in the analysis did not importantly change the reduced risk found with high zinc (OR adjusted for education = 0.41 95% CI 0.20–0.84).

**Table 2:** Age and main causes of infant deaths over 2 years by mean well concentration of zinc.

	Zinc (mg/L)							
	Low <0.02		Moderate 0.02 < 0.07			High 0.07 < 0.10		
	n *	OR	n	OR	95% CI	n	OR	95% CI
All deaths <12 months	305	1	292	1.04	0.70–1.56	337	0.95	0.56–1.63
Deaths <8 days	120	1	130	1.18	0.83–1.68	154	1.11	0.66–1.87
8–28 days	62	1	67	1.17	0.64–2.14	76	1.06	0.56–2.00
≥29 days	123	1	95	0.84	0.51–1.36	107	0.75	0.40–1.40
**Cause of Death:**								
‘prematurity’	74	1	83	1.22	0.73–2.02	105	1.23	0.69–2.20
pneumonia	164	1	133	0.88	0.52–1.48	161	0.85	0.51–1.41
diarrhoea	23	1	19	0.90	0.44–1.80	8	0.30	0.13–0.69
Number of births	9,657		8,909			11,178		
Number of upazillas	4		4			4		

* n = number of deaths.

## 4. Discussion

Zinc in well water appeared to confer no particular advantage in pregnancy (insofar as this is reflected in deaths from prematurity), consistent with the Cochrane review where zinc supplementation in pregnancy was seen only to reduce the number of preterm births; there was no advantage to neonatal health or survival [3]. Zinc easily crosses the placental barrier and maternal exposure during pregnancy, not only from drinking water but from food grown and cooked locally, will be available to the fetus. It is very unlikely, however, that the amounts ingested by the mother will approach those used in other communities as supplementation. In contrast, higher levels of zinc in well water did appear to confer some protection from deaths attributed to diarrhoea although, again, an infant’s intake of zinc (through breast milk or supplementary intake of food or water) would not approach that used as treatment after diarrhoea has developed (recommended as 20 mg/day for 10–14 days) [5] or for diarrhoea prevention, where doses of from 0.7–35 mg/day have been used [6]. Even in water from the well with the highest concentration (0.48 mg/L) an infant taking 600 cc of water supplementary to breast milk would only get an additional 0.29 mg/day from this source. In the present study, we do not know whether the lower rate of deaths from diarrhoea represents fewer cases or a lower case-fatality rate, but by analogy with the effects of zinc supplementation as treatment for diarrhoea, it is presumably the latter, if indeed the effect is real. Allowance for possible confounding was limited by the inability to match birth and death records at the individual level and the allocation of mean upazilla zinc level (from 14 wells or less) to all women giving birth in that upazilla will certainly have given rise to misclassification tending to drive estimates of effect towards the null. The rate of infant deaths in this cohort, and possibly the proportion attributed to diarrhoea (5.4%) may be lower than in villages without long term NGO commitment, and this may limit the extent to which the observation can be generalized to other communities. Nevertheless, the effect observed was specific to diarrhoea and has some biological plausibility. Although four meta-analyses have attempted to look at the effect of preventive supplementation on all-cause mortality (and found, as here, only a small and non-significant reduction in risk) none has looked at diarrhoea deaths specifically [6]. If replicated in similar populations, this might suggest some benefit of continuous low level supplementation with zinc for infants susceptible to diarrhoea living in areas with low natural exposure through drinking water.
